# Efficient Generation of Genome-Modified Mice Using *Campylobacter jejuni*-Derived CRISPR/Cas

**DOI:** 10.3390/ijms18112286

**Published:** 2017-10-31

**Authors:** Wataru Fujii, Arisa Ikeda, Koji Sugiura, Kunihiko Naito

**Affiliations:** Department of Animal Resource Sciences, Graduate School of Agricultural and Life Sciences, The University of Tokyo, 1-1-1, Yayoi, Tokyo 113-8657, Japan; ikeda-arisa228@g.ecc.u-tokyo.ac.jp (A.I.); aks@mail.ecc.u-tokyo.ac.jp (K.S.)

**Keywords:** CRISPR/Cas, genome-modified mouse, *Campylobacter jejuni*

## Abstract

Mammalian zygote-mediated genome-engineering by Clustered Regularly Interspaced Short Palindromic Repeat (CRISPR)/Cas is currently used for the generation of genome-modified animals. Here, we report that a *Campylobacter jejuni*-derived orthologous CRISPR/Cas system recognizes a 5′-NNNVRYAC sequence as a protospacer-adjacent motif in mouse zygotes, and is applicable for efficient generation of knockout mice. Moreover, this novel CRISPR/Cas can be used for zygote-mediated knock-in at a unique locus, suggesting that this system could help to expand the feasibility of the zygote-mediated generation of genome-modified animals.

## 1. Introduction

Zygote-mediated genome engineering using engineered endonucleases is an efficient way to generate genome-modified animals, and is widely used in diverse mammalian species [1,2,3,4,5,6,7,8,9]. The Clustered Regularly Interspaced Short Palindromic Repeat (CRISPR)/Cas system is the most commonly used engineered endonuclease, and consists of Cas9 endonuclease protein and synthetic guide-RNA (gRNA) [10,11]. The Cas9-gRNA complex recognizes a specific DNA sequence containing a base-pairing region and a protospacer adjacent motif (PAM), and induces a DNA double-strand break (DSB) in the target locus. The repair of a DSB by error-prone non-homologous end-joining (NHEJ) is sometimes accompanied by indel mutations, resulting in disruption of the target gene or genome information [10,11].

*Streptococcus pyogenes* (Sp)-derived CRISPR/Cas, which recognizes the 5′-NGG sequence as PAM, is currently the most utilized engineered endonuclease [12,13]. Meanwhile, orthologous CRISPR/Cas systems have been reported in various kinds of prokaryotes, and some of these use intrinsic PAM sequences different from Sp-CRISPR/Cas system [14]. It is expected that these orthologous CRISPR/Cas systems will be used as engineered endonucleases for the modification of diverse loci. It has already been reported that several orthologous CRISPR/Cas are applicable in cellulo, including in mammalian zygotes [15,16,17].

A *Campylobacter jejuni* (Cj)-derived type II CRISPR system has been reported to be usable as an engineered endonuclease in vitro and in vivo [14,18,19]. However, the question of which PAM sequences are available for this Cj-CRISPR/Cas is still a controversial one. Fonfara et al. were the first to report that Cj-CRISPR/Cas could be used as an engineered endonuclease in vitro, and that it recognizes the 5′-NNNNACA sequence as a PAM [14]. However, two subsequent reports using in vitro assays proposed much more versatile sequences, i.e., 5′-NNNNRYAC (R; A/G and Y; C/T) [18] and 5′-NNNVRYM (V; A/C/G and M; A/C) [19], and these sequences are conflicting at the 4th to 8th positions. Although some of the sequences have already been verified in culture cells and animal models [18], the discrepancy of availability has not been adequately validated in cellulo. Therefore, to apply Cj-CRISPR/Cas to zygote-mediated genome modification, it is necessary to investigate the functional conditions, including the applicable PAM sequences.

In the present study, we attempted to apply the Cj-derived CRISPR/Cas system to zygote-mediated genome engineering, and successfully generated systemic knockout (KO) mice. In addition, we were able to apply the orthologous CRISPR/Cas to zygote-mediated knock-in (KI) at a locus whose canonical target was difficult to be designed by the conventional CRISPR/Cas system.

## 2. Results

### 2.1. Examination of PAM Sequences Applicable to Mouse Zygotes

At first, we generated a mouse-codon-optimized Cj-Cas9 expressing vector (Figure S1), and the protein expression was confirmed by immunoblotting using HEK293 cells (Figure S2), in order to investigate PAM sequences that could be used for zygote-mediated genome-modification with the Cj-CRISPR/Cas system. The gRNAs were designed at the tyrosinase and Rosa26 loci (Table 1 and Figures S1 and S3), and each was microinjected into mouse zygotes together with mouse codon-optimized synthetic Cj-Cas9 mRNA. Mutagenesis efficiencies were then evaluated at the blastocyst stage. More than 90% of the 2-cell stage embryos reached the blastocyst stage regardless of which gRNA was used (Figure S3C).

Our initial examination of the 5′-NNNVACAC sequence (T1, T2, and R1 in Table 1), which is common to all previously proposed PAM sequences [14,18,19], showed that all embryos injected with each gRNA had indel mutations at the target loci (Table 1). To examine the availability of more versatile sequences at the 5th and 6th base positions, which were common in two recent reports [18,19], we designed gRNAs using 5′-NNNVGCAC (R2 in Table 1), 5′-NNNVATAC (T3 in Table 1), and 5′-NNNVGTAC (T4 in Table 1) as PAMs. These gRNAs were available to induce the indels, although the mutagenesis efficiencies were lower than that of 5′-NNNVACAC (Table 1). We next examined the feasibility of the 7th and 8th positions, which have been allowed M (A/C) and N (A/C/T/G), respectively, in previous reports [19]. However, indel mutation was not observed in any embryos injected with the gRNAs designed for 5′-NNNVRYCC (T5, T6, and T7) and 5′-NNNVRYAN (T8 and T9) (Table 1). Further, the 4th position of a functional PAM was changed to T (T10, T11, T12, T13, and R3); this, it has been suggested [19], interrupts its recognition as a PAM. As a result, mutation induction was not observed in any embryos (Table 1). These results suggested that zygote-mediated genome-modification by Cj-CRISPR/Cas is possible when the 5′-NNNVRYAC sequence is used as the PAM.

### 2.2. Generation of KO Mice Using Cj-CRISPR/Cas

**Figure 1:**
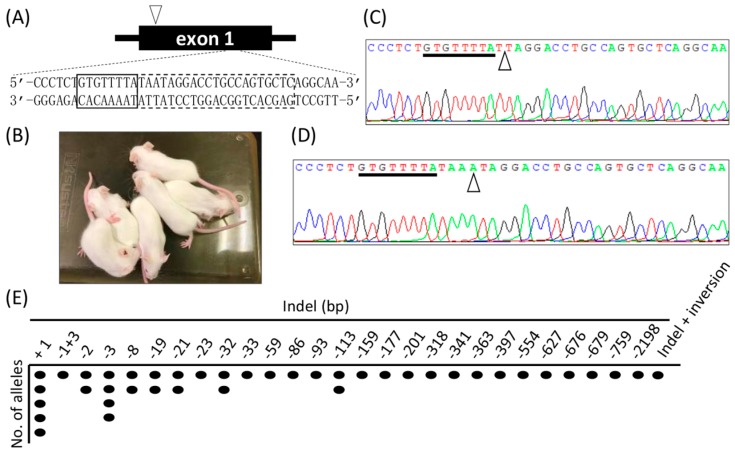
Generation of tyrosinase-KO mice using Cj-CRISPR/Cas. (**A**) Schematics of the target locus in tyrosinase. The box outlined with a dotted line indicates the recognition sequence by gRNA, and that outlined with a solid-line indicates PAM. An arrowhead points to the start codon locus of tyrosinase. (**B**) Some of the offspring by embryo transfer of injected zygotes. (**C**) Waveform data of deletion mutation and (**D**) insertion mutation in the obtained pups. PAM and mutation sites are indicated by underline and arrowheads. (**E**) Suggested mutation patterns in all the offspring.

Next, we attempted to generate KO mice using Cj-CRISPR/Cas. Cas9 mRNA and gRNA T1 (Table 1 and Figure 1A) were injected into the C57BL/6NCr zygotes, 72 two-cell embryos were transferred to recipients, and 20 offspring were obtained. All of the obtained pups had white hair (Figure 1B), suggesting tyrosinase deficiency. Induced mutations were detected using tail-tip-derived genomic DNA, and all of the pups had inserted or deleted mutations at the target locus (Figure 1C–E). In addition, we examined the off-target effects at three loci, with the result that no indel mutation was observed in the pups (Table S3). These results suggested that Cj-CRISPR/Cas is appropriate for the efficient generation of accurate genome-modified mouse via zygotes.

### 2.3. Zygote-Mediated KI by Cj-CRISPR/Cas

**Figure 2:**
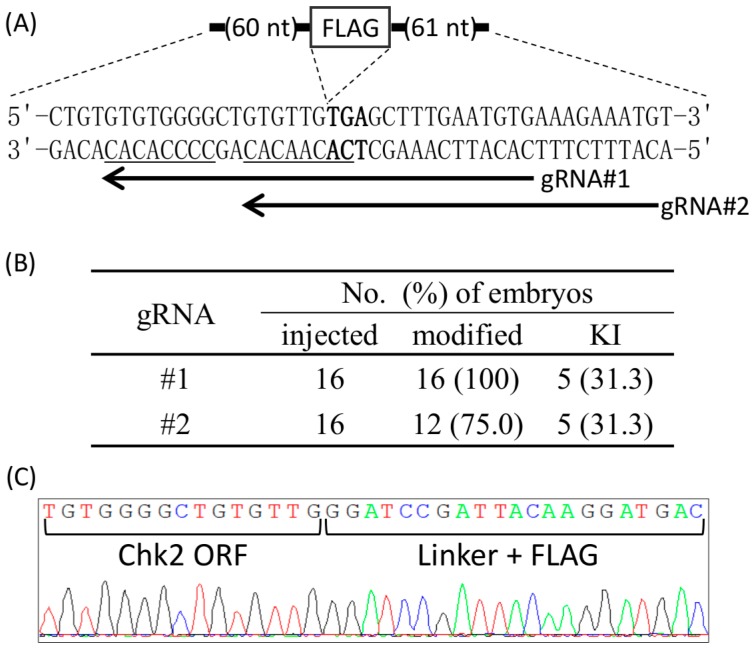
Zygote-mediated KI at the C-terminal region of Chk2 using Cj-CRISPR/Cas. (**A**) Schematics of the target sequence. Arrows and underlined sequences indicate the target loci of each gRNAs and its PAM, respectively. The stop codon of Chk2 is shown in bold. The DNA sequence of ssODN is shown in Table S1. (**B**) Generation efficiencies of genome-modified embryos. (**C**) Waveform data of the target locus from an embryo modified using gRNA #2. ORF: open reading frame, FLAG: DYKDDDDK epitope tag sequence.

We next examined whether Cj-CRISPR/Cas could be applied to KI via zygotes at a locus that is difficult to target with conventional CRISPR/Cas. The KI using Sp-CRISPR/Cas together with ssODN would not be available at C-terminal of mouse checkpoint kinase 2 (Chk2), because there is no canonical-PAM sequence around the stop codon. In contrast, the target sequences of Cj-CRISPR/Cas could be designed to overlap the stop codon of Chk2. Therefore, we designed two gRNAs, and each was microinjected together with Cas9 mRNA and ssODN-encoding Flag-tag sequence and homology arms (Figure 2A). The target locus of each embryo was then examined at the blastocyst stage. Mutations were observed in all of the embryos injected with #1 gRNA, and 12 of 16 embryos injected with #2 gRNA. Furthermore, accurate KI of the exogenous Flag-tag sequence was observed in 5 embryos by use of either gRNAs (Figure 2B). In particular, two embryos generated by injection of #1 gRNA showed only KI sequences without wildtype or KO sequence (Figure 2C), suggesting bi-allelic KI. These results suggested that Cj-CRISPR/Cas could be used for zygote-mediated KI of unique sequences.

## 3. Discussion

In the present study, we attempted to apply *C. jejuni*-derived CRISPR/Cas to zygote-mediated genome-modification in mice. Our experiments suggested that 5′-NNNVRYAC is suitable for use as a PAM for mutagenesis in zygotes, and Cj-CRISPR/Cas is capable of zygote-mediated generation of genome-modified animals, as well as previously reported engineered endonucleases. Cj-Cas9 is known as one of the smallest Cas9 orthologs [14], which is advantageous for use as an all-in-one adeno-associated virus vector in in vivo gene therapy [18]. In such applications, it is necessary to clarify the efficient, usable PAM sequences in mammalian cells. In this study, we evaluated the practicality of the PAM sequence proposed by previous biochemical assays for in cellulo applications. It was expected that our findings would be informative for the application of Cj-CRISPR/Cas to various types of mammalian cells in vivo and in vitro.

The PAM sequence proposed in the present study suggests that Cj-CRISPR/Cas has a potential to compensate for a part of the loci, which could not be covered by the previously applied orthologs including conventional CRISPR/Cas, indicated that the zygote-mediated genome-modification is possible at much diverse target loci. Particularly in the case of KI using ssODN, it is necessary to design a gRNA near the target locus, so the designable loci of gRNA are limited [20]. This study essentially targeted the C-terminal region of Chk2, where Sp-CRISPR/Cas is difficult to be applied as a canonical designing, and accurate KI was achieved by Cj-CRISPR/Cas. It is expected that Cj-CRISPR/Cas would be applicable to such KI or nucleotide substitutions.

In this study, we did not observe the versatility of the 7th and 8th positions of PAM in zygotes despite previous suggestions by biochemical screening assays. Meanwhile, the substitution of the 4th position to T inhibited the function of PAM, just as it did in vitro. However, we could not conclude that such sequences are not recognized as PAM by Cj-CRISPR/Cas in zygotes, because the evaluated loci were limited in the present study. It is expected that the accuracy of available PAM sequence information will be improved by further evaluations in additional loci. Understanding the PAM sequences available for in cellulo analysis is important for predicting off-target candidate sequences, as well as for target design. It is necessary to thoroughly examine various candidate sequences proposed by in vitro assay through further applications of Cj-CRISPR/Cas in mammalian zygotes or diverse kinds of cells.

## 4. Materials and Methods

### 4.1. Ethics Statement

All animal care and experiments conformed to the Guidelines for Animal Experiments of The University of Tokyo and were approved by the Animal Research Committee of The University of Tokyo (P17-047, 5 June 2017).

### 4.2. Construction of Cas9-and gRNA-plasmid DNA

The amino acid sequence of *C. jejuni* Cas9 was obtained from the National Center for Biotechnology Information (accession no: WP_002851159), and the mouse codon-optimized nucleotide sequence was designed using the IDT Codon Optimization Tool (Available online: https://sg.idtdna.com/CodonOpt). The DNA sequence of the Cas9 ORF, which contains the Flag-tag sequence at the N-terminal and an SV40 nuclear localization signal at each of the N- and C-terminals, was synthesized by Thermo Fisher Scientific (Waltham, MA, USA), and the DNA fragment was inserted into the NotI to ClaI site of the pCAG-T3-hCAS9-pA plasmid (Addgene #48625 [9]), which codes the T3 promoter, Tbpl1 3′UTR, and 95 nucleotides of polyadenine. The constructed plasmid vector was sequenced using a commercial sequencing kit (Applied Biosystems, Foster City, CA, USA) and a DNA sequencer (Applied Biosystems, Foster City, CA, USA) according to the manufacturer’s instructions.

The platform vector for *C. jejuni* gRNA was constructed by overlap-PCR using the primers shown in Table S1 and sub-cloned using a pGEM-T Easy system (Promega, Madison, WI, USA). The vector was sequenced as described above. Each gRNA vector was constructed by ligation of the BsmBI-digested platform vector with an annealed dsDNA fragment encoding the corresponding DNA recognition sequence.

All of the sequence information is provided in Figure S1.

### 4.3. DNA Transfection and Immunoblotting to Validate Cj-Cas9 Expression

HEK 293 cells (2 × 10^5^) were seeded in each well of a 24-well plate coated with 0.2% gelatin solution, and cultured in Dulbecco’s modified Eagle’s medium (DMEM) supplemented with 10% FBS at 37 °C with 5% CO_2_ in air. The constructed Cas9-expressing vector was transfected into the HEK293 cells by Lipofectamine LTX reagent (Life Technologies, Carlsbad, CA, USA) according to the manufacturer’s protocols. The transfected cells were harvested 72 h after transfection, and suspended in Laemmli-buffer. Western blot analysis was performed according to the process described in our previous report [9]. The signals were detected using an Immunostar LD Kit (Wako, Tokyo, Japan) and a C-DiGit scanner (LI-COR, Lincoln, NE, USA).

### 4.4. In Vitro Transcription of Cas9 mRNA and gRNAs

The in vitro synthesis of Cas9 mRNA and gRNA was performed as described previously [21]. The RNA transcripts were precipitated with absolute Figureethanol, washed, and resuspended in RNase-free water (Gibco, Grand Island, NY, USA). The RNA solutions were stored at −80 °C until use.

### 4.5. In Vitro Fertilization

Sexually immature female C57BL/6NCr mice (3-weeks old) were superovulated by an intraperitoneal injection of 7.5 IU equine chorionic gonadotropin (eCG), followed by 7.5-IU human chorionic gonadotropin (hCG) 48 h later. Cumulus-oocyte complexes were collected from oviductal ampulla 14 h after the hCG injection, and were co-cultured with sperm collected from the cauda epididymidis of mature male C57BL/6NCr mice (>9 weeks old) in HTF medium. After 5 h of co-culture, pronuclei-formed zygotes were put into KSOMaa.

### 4.6. Microinjection and Embryo-Transfer

The microinjection was performed using a microinjector (Narishige, Tokyo, Japan)-equipped microscope. Approximately 4 pL of RNA solution, containing 100 ng/μL of Cas9 mRNA and 20 ng/μL of gRNA (with 100 ng/μL ssODN as shown in Table S1 only for the KI experiment), was injected into the cytoplasm of each zygote using continuous pneumatic pressure. The injected zygotes were cultured in KSOMaa, and after 24 h, two-cell embryos were transferred into the oviduct of 0.5 days post-coitum pseudopregnant ICR females; alternatively, after 96 h, blastocysts were subjected to genotyping.

### 4.7. Detection of Induced Mutations

The genome DNA was extracted from the blastocyst embryos or the tail tips of the pups according to our previous report [21], and then subjected to PCR for on-target locus and off-target loci (Table S3) using the primer sets shown in Table S2. The potential off-target loci for Tyr gRNA T1 were searched using a Genome-wide tag scanner [22]. Briefly, a partial sequence of T1 target sequence (5′-CTGGCAGGTCCTATTANNNNACAC) with two mismatches was used to search, and three of the resulted loci, which have 5′-NNNNACAC as PAM, were adopted as the potential off-target loci (Table S3). The PCR products were purified by agarose gel electrophoresis, and extracted fragments were directly sequenced as described above. The predictive mutation pattern of each allele was analyzed by sequence data as described above.

## Figures and Tables

**Table 1:** Mutagenesis efficiencies by each gRNA.

gRNA *	PAM Sequence						Mutated/Total
	1st-3rd	4th	5th	6th	7th	8th	(%)
**Reported PAM**							
5′-NNNNACA [14]	NNN	all	A	C	A	all	−
5′-NNNNRYAC [18]	NNN	all	A/G	C/T	A	C	−
5′-NNNVRYM [19]	NNN	no T	A/G	C/T	A/C	all	−
**common in all**	NNN	no T	A	C	A	C	
T1	TAA	A	A	C	A	C	16/16 (100)
T2	TGT	G	A	C	A	C	16/16 (100)
R1	TGC	A	A	C	A	C	16/16 (100)
**5th and/or 6th change**	NNN	no T	G	T	A	C	
T3	GGC	A	A	T	A	C	12/16 (75.0)
T4	TGA	A	G	T	A	C	7/16 (43.8)
R2	GGC	C	G	C	A	C	13/16 (81.3)
**7th change**	NNN	no T	A	C	C	C	
T5	ATC	C	A	C	C	C	0/16 (0)
T6	CAT	C	A	C	C	C	0/16 (0)
T7 (also 5th)	CCT	G	G	C	C	C	0/16 (0)
**8th change**	NNN	no T	A	C	A	no C	
T8	AAA	C	A	C	A	G	0/16 (0)
T9	AAG	A	A	C	A	T	0/16 (0)
**4th change**	NNN	T	A	C	A	C	
T10	TTT	T	A	C	A	C	0/16 (0)
T11 (also 5th)	CAG	T	G	C	A	C	0/16 (0)
T12 (also 5th)	TGG	T	G	C	A	C	0/16 (0)
T13 (also 5/6th)	AAT	T	G	T	A	C	0/16 (0)
R3	TTT	T	A	C	A	C	0/16 (0)

* Target sequences and schematics of designed loci are shown in Figure S3.

## Supplementary Materials

Supplementary materials can be found at www.mdpi.com/1422-0067/18/11/2286/s1.

## Abbreviations

CRISPR	Clustered Regularly Interspaced Short Palindromic Repeat
Cas	CRISPR-Associated
PAM	protospacer adjacent motif
gRNA	guide-RNA
DSB	DNA double-strand break
NHEJ	non-homologous end-joining
Sp	*Streptococcus pyogenes*
Cj	*Campylobacter jejuni*
KO	knockout
KI	knock-in
ssODN	single-strand oligodeoxynucleotide
Chk2	checkpoint kinase 2
DMEM	Dulbecco’s modified Eagle’s medium
FBS	fetal bovine serum
eCG	equine chorionic gonadotropin
hCG	human chorionic gonadotropin

## References

[1] Meyer M., de Angelis M.H., Wurst W., Kühn R. (2010). Gene targeting by homologous recombination in mouse zygotes mediated by zinc-finger nucleases. Proc. Natl. Acad. Sci. USA.

[2] Geurts A.M., Cost G.J., Freyvert Y., Zeitler B., Miller J.C., Choi V.M., Jenkins S.S., Wood A., Cui X., Meng X. (2009). Knockout rats via embryo microinjection of zinc-finger nucleases. Science.

[3] Sung Y.H., Baek I.J., Kim D.H., Jeon J., Lee J., Lee K., Jeong D., Kim J.S., Lee H.W. (2013). Knockout mice created by TALEN-mediated gene targeting. Nat. Biotechnol..

[4] Shen B., Zhang J., Wu H., Wang J., Ma K., Li Z., Zhang X., Zhang P., Huang X. (2013). Generation of gene-modified mice via Cas9/RNA-mediated gene targeting. Cell Res..

[5] Wang H., Yang H., Shivalila C.S., Dawlaty M.M., Cheng A.W., Zhang F., Jaenisch R. (2013). One-step generation of mice carrying mutations in multiple genes by CRISPR/Cas-mediated genome engineering. Cell.

[6] Yang D., Xu J., Zhu T., Fan J., Lai L., Zhang J., Chen Y.E. (2014). Effective gene targeting in rabbits using RNA-guided Cas9 nucleases. J. Mol. Cell Biol..

[7] Hai T., Teng F., Guo R., Li W., Zhou Q. (2014). One-step generation of knockout pigs by zygote injection of CRISPR/Cas system. Cell Res..

[8] Niu Y., Shen B., Cui Y., Chen Y., Wang J., Wang L., Kang Y., Zhao X., Si W., Li W. (2014). Generation of gene-modified cynomolgus monkey via Cas9/RNA-mediated gene targeting in one-cell embryos. Cell.

[9] Fujii W., Kawasaki K., Sugiura K., Naito K. (2013). Efficient generation of large-scale genome-modified mice using gRNA and CAS9 endonuclease. Nucleic Acids Res..

[10] Sander J.D., Joung J.K. (2014). CRISPR-Cas systems for editing, regulating and targeting genomes. Nat. Biotechnol..

[11] Hsu P.D., Lander E.S., Zhang F. (2014). Development and applications of CRISPR-Cas9 for genome engineering. Cell.

[12] Cong L., Ran F.A., Cox D., Lin S., Barretto R., Habib N., Hsu P.D., Wu X., Jiang W., Marraffini L.A. (2013). Multiplex genome engineering using CRISPR/Cas systems. Science.

[13] Mali P., Yang L., Esvelt K.M., Aach J., Guell M., DiCarlo J.E., Norville J.E., Church G.M. (2013). RNA-guided human genome engineering via Cas9. Science.

[14] Fonfara I., Le Rhun A., Chylinski K., Makarova K.S., Lécrivain A.L., Bzdrenga J., Koonin E.V., Charpentier E. (2014). Phylogeny of Cas9 determines functional exchangeability of dual-RNA and Cas9 among orthologous type II CRISPR-Cas systems. Nucleic Acids Res..

[15] Esvelt K.M., Mali P., Braff J.L., Moosburner M., Yaung S.J., Church G.M. (2013). Orthogonal Cas9 proteins for RNA-guided gene regulation and editing. Nat. Methods.

[16] Hirano H., Gootenberg J.S., Horii T., Abudayyeh O.O., Kimura M., Hsu P.D., Nakane T., Ishitani R., Hatada I., Zhang F. (2016). Structure and Engineering of Francisella novicida Cas9. Cell.

[17] Fujii W., Kakuta S., Yoshioka S., Kyuwa S., Sugiura K., Naito K. (2016). Zygote-mediated generation of genome-modified mice using Streptococcus thermophilus 1-derived CRISPR/Cas system. Biochem. Biophys. Res. Commun..

[18] Kim E., Koo T., Park S.W., Kim D., Kim K., Cho H.Y., Song D.W., Lee K.J., Jung M.H., Kim S. (2017). In vivo genome editing with a small Cas9 orthologue derived from *Campylobacter jejuni*. Nat. Commun..

[19] Yamada M., Watanabe Y., Gootenberg J.S., Hirano H., Ran F.A., Nakane T., Ishitani R., Zhang F., Nishimasu H., Nureki O. (2017). Crystal Structure of the Minimal Cas9 from *Campylobacter jejuni* Reveals the Molecular Diversity in the CRISPR-Cas9 Systems. Mol. Cell.

[20] Fujii W. (2017). Generation of Knock-in Mouse by Genome Editing. Methods Mol. Biol..

[21] Fujii W., Kano K., Sugiura K., Naito K. (2013). Repeatable construction method for engineered zinc finger nuclease based on overlap extension PCR and TA-cloning. PLoS ONE.

[22] Iseli C., Ambrosini G., Bucher P., Jongeneel C.V. (2007). Indexing strategies for rapid searches of short words in genome sequences. PLoS ONE.

