# Supplementary material for: Efficient Generation of Genome-Modified Mice Using Campylobacter jejuni-Derived CRISPR/Cas
Source: Int J Mol Sci. 2017 Oct 31;18(11):2286. doi: 10.3390/ijms18112286 (PMC5713256; doi:10.3390/ijms18112286)
Supplement: Supplementary file 1 [file ijms-18-02286-s001.pdf]

Efficient generation of genome-modified mice using Campylobacter jejuni-derived CRISPR/Cas

Wataru Fujii, Arisa Ikeda, Koji Sugiura, Kunihiko Naito

**Supplementary Information**


**Supplementary Figure 1** Sequences of Cj-Cas9 and gRNAs.

**Supplementary Figure 2** Confirmation of Cj-Cas9 expression by immunoblotting.

**Supplementary Figure 3** Schematics of target loci of each gRNAs.

**Supplementary Table 1** Primer sets for synthesis of Cj-gRNA template vectors and ssODN.

**Supplementary Table 2** Primer sets for genomic PCR.

**Supplementary Table 3** On-target and off-target loci of *Tyrosinase*.

**Supplementary Figure 1. Sequences of Cj-Cas9 and gRNAs.**

>DNA sequence of Cj-Cas9 vector (from T3 promoter to SphI site)

GAAATTAACCCTCACTAAAGGGAACAAAAGCTGGAGCTCCACCGCGGTGGCGGCCGCGGGAATTCGA
TTCCACCATGGACTATAAGGACGACGACGATAAGATAATGGCCCCTAAGAAGAAAAGAAAGGTGGG
AtccATGGCCCGCATTCTTGCTTTCGATATTGGCATCAGCTCAATAGGATGGGCATTTTCCGAGAATGA
CGAATTGAAGGACTGCGGAGTTAGGATCTTCACTAAAGTAGAGAACCCTAAGACTGGTGAATCCCTG
GCACTGCCCAGAAGACTGGCTAGGAGCGCACGGAAGCGACTCGCTCGCAGAAAGGCAAGACTTAAT
CACCTTAAACACCTTATTGCAAACGAGTTCAAGCTCAATTACGAAGATTATCAGTCTTTTGACGAAAG
CCTTGCCAAGGCCTACAAAGGTAGTCTCATCAGCCCCTACGAACTCAGGTTCAGGGCACTGAATGAA
CTCCTGTCCAAGCAGGATTTCGCAAGAGTCATACTTCACATAGCTAAACGGAGGGGCTATGATGACA
TAAAGAACTCCGACGACAAGGAAAAAGGGGCTATACTTAAGGCAATCAAACAGAACGAAGAGAAAC
TTGCTAACTACCAATCAGTAGGAGAATACCTGTATAAGGAATATTTTCAGAAGTTTAAGGAAAACTCC
AAGGAATTCACTAACGTTCGAAACAAGAAGGAGTCTTATGAACGCTGCATCGCACAGAGTTTCTTGA
AGGATGAGCTGAAACTTATATTCAAAAAGCAAAGGGAGTTCGGATTCTCCTTTAGCAAAAAATTTGA
AGAGGAGGTGTTGAGCGTCGCTTTTTACAAACGGGCACTCAAAGACTTTTCACATCTGGTTGGCAATT
GCTCTTTCTTTACCGATGAGAAACGCGTACCTAAGAATAGTCCACTCGCTTTTATGTTTGTCGCTTTGA
CACGAATTATAAACCTGCTGAATAACCTTAAAAATACAGAGGGCATCCTGTACACAAAGGATGATCT
CAATGCTCTGTTGAATGAAGTTCTCAAGAACGGTACTCTGACTTATAAACAAACAAAGAAGCTGCTC
GGTCTGTCAGACGATTATGAGTTTAAGGGCGAAAAGGGGACCTACTTTATCGAGTTTAAGAAGTATA
AAGAATTCATCAAAGCCCTGGGAGAGCATAACTTGTCACAAGATGATCTTAATGAGATTGCAAAAGA
TATAACCCTCATAAAGGATGAGATTAAACTGAAAAAGGCCCTCGCCAAGTACGATCTTAATCAGAAC
CAAATTGACTCATTGAGCAAGCTGGAATTCAAAGATCACCTGAACATTTCATTTAAGGCCCTCAAGCT
TATTACACCCTTGATGCTGGAAGGCAAAAAATATGACGAAGCTTGCAACGAGCTCAATCTGAAAGTC
GCCATAAACGAGGATAAAAAAGATTTCCTTCCCGCATTTAACGAGACCTATTACAAGGATGAAGTGA
CTAATCCCGTGGTTCTTCGAGCAATAAAGGAATACAGGAAAGTTCTTAATGCACTCCTCAAAAAATAT
GGCAAAGTACATAAAATTAACATAGAGCTCGCCCGAGAGGTAGGAAAGAATCATTCCCAAAGGGCA
AAAATCGAAAAAGAGCAAAATGAAAATTACAAGGCAAAGAAGGATGCCGAGCTCGAGTGCGAAAAA
CTGGGGCTGAAGATCAATTCCAAGAACATATTGAAGCTGCGACTCTTCAAGGAACAGAAGGAATTTT
GTGCTTACAGCGGAGAAAAAATAAAAATAAGCGATCTGCAAGATGAAAAGATGCTCGAGATCGACC
ACATCTACCCATATTCAAGATCTTTCGACGACTCCTACATGAATAAAGTCCTCGTTTTTACCAAACAG
AATCAAGAGAAGCTCAACCAGACCCCCTTTGAGGCCTTCGGCAACGACAGTGCAAAGTGGCAAAAAA
TAGAAGTCCTCGCTAAAAATCTCCCCACAAAAAAACAAAAGAGGATATTGGACAAGAACTATAAAG
ATAAGGAGCAAAAAAACTTTAAAGACAGGAATCTTAACGACACTAGATACATCGCTAGACTGGTATT
GAATTATACCAAAGACTACCTGGACTTTCTTCCACTGAGCGACGACGAAAACACAAAACTCAACGAC
ACACAGAAAGGTAGTAAGGTACATGTTGAGGCCAAAAGTGGAATGTTGACTTCAGCCCTTCGACATA
CCTGGGGTTTTTCTGCTAAAGATCGCAATAACCACCTTCACCATGCCATTGATGCTGTTATTATTGCAT
ACGCTAACAACTCAATAGTTAAGGCTTTCTCTGATTTTAAGAAAGAACAGGAAAGCAATTCTGCTGA

GCTCTATGCAAAAAAGATCAGCGAGTTGGATTACAAGAACAAGAGGAAATTTTTGAGCCCTTTTCC
GGATTCAGACAAAAGGTCTTGGACAAAATTGATGAGATTTTTGTGTCAAAGCCTGAGAGAAAGAAGC
CTAGTGGAGCCCTGCACGAGGAAACCTTTAGGAAGGAGGAGGAATTTTATCAAAGTTACGGTGGTAA
AGAGGGTGTTCTTAAAGCACTCGAGCTTGGAAAGATCCGGAAGGTGAACGGGAAGATAGTCAAAAA
TGGGGACATGTTCCGGGTCGATATTTTTAAGCACAAGAAGACAAACAAGTTCTACGGTGTACCCATAT
ACACCATGGATTTTGCTCTTAAAGTATTGCCAAATAAGGCTGTGGCTAGAAGCAAGAAAGGAGAAAT
CAAGGACTGGATACTCATGGATGAAAACTACGAATTCTGCTTCTCACTTTACAAGGACTCCCTCATTC
TCATCCAAACCAAAGATATGCAGGAACCTGAGTTCGTTTACTATAATGCTTTTACCAGCTCTACCGTG
TCCCTTATCGTCTCCAAGCACGACAATAAATTCGAAACACTGTCTAAGAATCAAAAAATTTTGTTTAA
GAATGCTAACGAGAAGGAGGTGATTGCAAAGAGCATTGGCATTCAAAACCTGAAAGTGTTTGAAAAA
TACATCGTTTCAGCACTGGGAGAAGTAACAAAGGCTGAATTTAGGCAAAGAGAGGACTTCAAGAAAG
GATCCATGGCTCCTAAGAAAAAACGGAAGGTTTGAATCGATGGTACCTATGGGCACCAAAGAACCTG
TAAACGTTATCTTTTTAAATTGAATGTGCACAAATAAAAGTTTGGAAAAGAAAAAAAAAAAAAAAAA
AAAAAAAAAAAAAAAAAAAAAAAAAAAAAAAAAAAAAAAAAAAAAAAAAAAAAAAAAAAAAAAA
AAAAAAAAAAAACCCTCGAGGTAGCATGC

>Amino acid sequence of Cj-Cas9
MDYKDDDDKIMAPKKKRKVGSMARILAFDIGISSIGWAFSENDELKDCGVRIFTKVENPKTGESLALPRRL
ARSARKRLARRKARLNHLKHLIANEFKLNYEDYQSFDESLAKAYKGSLISPYELRFRALNELLSKQDFARV
ILHIAKRRGYDDIKNSDDKEKGAILKAIKQNEEKLANYQSVGEYLYKEYFQKFKENSKEFTNVRNKKESY
ERCIAQSFLKDELKLIFKKQREFGFSFSKKFEEEVLSVAFYKRALKDFSHLVGNCSFFTDEKRVPKNSPLAF
MFVALTRIINLLNNLKNTEGILYTKDDLNALLNEVLKNGTLTYKQTKKLLGLSDDYEFKGEKGTYFIEFKK
YKEFIKALGEHNLSQDDLNEIAKDITLIKDEIKLKKALAKYDLNQNQIDSLSKLEFKDHLNISFKALKLITPL
MLEGKKYDEACNELNLKVAINEDKKDFLPAFNETYYKDEVTNPVVLRAIKEYRKVLNALLKKYGKVHKI
NIELAREVGKNHSQRAKIEKEQNENYKAKKDAELECEKLGLKINSKNILKLRLFKEQKEFCAYSGEKIKISD
LQDEKMLEIDHIYPYSRSFDDSYMNKVLVFTKQNQEKLNQTPFEAFGNDSAKWQKIEVLAKNLPTKKQK
RILDKNYKDKEQKNFKDRNLNDTRYIARLVLNYTKDYLDFLPLSDDENTKLNDTQKGSKVHVEAKSGML
TSALRHTWGFSAKDRNNHLHHAIDAVIIAYANNSIVKAFSDFKKEQESNSAELYAKKISELDYKNKRKFFE
PFSGFRQKVLDKIDEIFVSKPERKKPSGALHEETFRKEEEFYQSYGGKEGVLKALELGKIRKVNGKIVKNG
DMFRVDIFKHKKTNKFYGVPIYTMDFALKVLPNKAVARSKKGEIKDWILMDENYEFCFSLYKDSLILIQTK
DMQEPEFVYYNAFTSSTVSLIVSKHDNKFETLSKNQKILFKNANEKEVIAKSIGIQNLKVFEKYIVSALGEV
TKAEFRQREDFKKGSMAPKKKRKV

>gRNA template vector (from T3 promoter to DraI site)
GAAATTAACCCTCACTAAAGGAGAGACGATCGTCTCTGTTTTAGTCCCTGAAAAGGGACTAAAATAA
AGAGTTTGCGGGACTCTGCGGGGTTACAATCCCCTAAAACCGCTTTTAAA

>gRNA T1 (from T3 promoter to DraI site)
GAAATTAACCCTCACTAAAGGAGCACTGGCAGGTCCTATTAGTTTTAGTCCCTGAAAAGGGACTAA
AATAAAGAGTTTGCGGGACTCTGCGGGGTTACAATCCCCTAAAACCGCTTTTAAA

>gRNA T2 (from T3 promoter to DraI site)
GAAATTAACCCTCACTAAAGGGGTTGCTGGAAAAGAAGTCGTTTTAGTCCCTGAAAAGGGACTAAA
ATAAAGAGTTTGCGGGACTCTGCGGGGTTACAATCCCCTAAAACCGCTTTTAAA

>gRNA T3 (from T3 promoter to DraI site)
GAAATTAACCCTCACTAAAGGAGATCTGGAAACTCCACAGAAGTTTTAGTCCCTGAAAAGGGACTA
AAATAAAGAGTTTGCGGGACTCTGCGGGGTTACAATCCCCTAAAACCGCTTTTAAA

>gRNA T4 (from T3 promoter to DraI site)
GAAATTAACCCTCACTAAAGGAGCATGAAAATGTGGCTGCGTTTTAGTCCCTGAAAAGGGACTAAA
ATAAAGAGTTTGCGGGACTCTGCGGGGTTACAATCCCCTAAAACCGCTTTTAAA

>gRNA T5 (from T3 promoter to DraI site)
GAAATTAACCCTCACTAAAGGAGGGCCAGGACTCACGGTCGTTTTAGTCCCTGAAAAGGGACTAAA
ATAAAGAGTTTGCGGGACTCTGCGGGGTTACAATCCCCTAAAACCGCTTTTAAA

>gRNA T6 (from T3 promoter to DraI site)
GAAATTAACCCTCACTAAAGGAAAGCTGGCCGCAGGGACTCCGTTTTAGTCCCTGAAAAGGGACTA
AAATAAAGAGTTTGCGGGACTCTGCGGGGTTACAATCCCCTAAAACCGCTTTTAAA

>gRNA T7 (from T3 promoter to DraI site)
GAAATTAACCCTCACTAAAGGGGTGGATGACCGTGAGTGTTTTAGTCCCTGAAAAGGGACTAAAAT
AAAGAGTTTGCGGGACTCTGCGGGGTTACAATCCCCTAAAACCGCTTTTAAA

>gRNA T8 (from T3 promoter to DraI site)
GAAATTAACCCTCACTAAAGGCACTGGCAGGTCCTATTATAGTTTTAGTCCCTGAAAAGGGACTAAA
ATAAAGAGTTTGCGGGACTCTGCGGGGTTACAATCCCCTAAAACCGCTTTTAAA

>gRNA T9 (from T3 promoter to DraI site)
GAAATTAACCCTCACTAAAGGAAGGCAATACAAAACAGCCGTTTTAGTCCCTGAAAAGGGACTAAA
ATAAAGAGTTTGCGGGACTCTGCGGGGTTACAATCCCCTAAAACCGCTTTTAAA

>gRNA T10 (from T3 promoter to DraI site)
GAAATTAACCCTCACTAAAGGCATCAAATACTCTCAGCCTGGTTTTAGTCCCTGAAAAGGGACTAAA
ATAAAGAGTTTGCGGGACTCTGCGGGGTTACAATCCCCTAAAACCGCTTTTAAA

>gRNA T11 (from T3 promoter to DraI site)
GAAATTAACCCTCACTAAAGGCCTGCCAGGATATCCTTCTGTCGTTTTAGTCCCTGAAAAGGGACTA
AAATAAAGAGTTTGCGGGACTCTGCGGGGTTACAATCCCCTAAAACCGCTTTTAAA

>gRNA T12 (from T3 promoter to DraI site)
GAAATTAACCCTCACTAAAGGAAGGGGAACTGAGGTCCAGAGTTTTAGTCCCTGAAAAGGGACTAA
AATAAAGAGTTTGCGGGACTCTGCGGGGTTACAATCCCCTAAAACCGCTTTTAAA

>gRNA T13 (from T3 promoter to DraI site)
GAAATTAACCCTCACTAAAGGAAGTTTGGATTTGGGGGCCCAGTTTTAGTCCCTGAAAAGGGACTA
AAATAAAGAGTTTGCGGGACTCTGCGGGGTTACAATCCCCTAAAACCGCTTTTAAA

>gRNA R1 (from T3 promoter to DraI site)
GAAATTAACCCTCACTAAAGGAGAACTCCCAGAAAGGTATGTTTTAGTCCCTGAAAAGGGACTAAA
ATAAAGAGTTTGCGGGACTCTGCGGGGTTACAATCCCCTAAAACCGCTTTTAAA

>gRNA R2 (from T3 promoter to DraI site)
GAAATTAACCCTCACTAAAGGAGTGGAGTAGGCGGGGAGAAGTTTTAGTCCCTGAAAAGGGACTAA
AATAAAGAGTTTGCGGGACTCTGCGGGGTTACAATCCCCTAAAACCGCTTTTAAA

>gRNA R3 (from T3 promoter to DraI site)
GAAATTAACCCTCACTAAAGGTGGGAAGTCTTGTCCCTCCAAGTTTTAGTCCCTGAAAAGGGACTAA
AATAAAGAGTTTGCGGGACTCTGCGGGGTTACAATCCCCTAAAACCGCTTTTAAA

>gRNA #1 for Chk2 (from T3 promoter to DraI site)
GAAATTAACCCTCACTAAAGGCACATTCAAAGCTCACAACACAGGTTTTAGTCCCTGAAAAGGGACT
AAAATAAAGAGTTTGCGGGACTCTGCGGGGTTACAATCCCCTAAAACCGCTTTTAAA

>gRNA #2 for Chk2 (from T3 promoter to DraI site)
GAAATTAACCCTCACTAAAGGCATTTCTTTCACATTCAAAGCTGTTTTAGTCCCTGAAAAGGGACTAA
AATAAAGAGTTTGCGGGACTCTGCGGGGTTACAATCCCCTAAAACCGCTTTTAAA

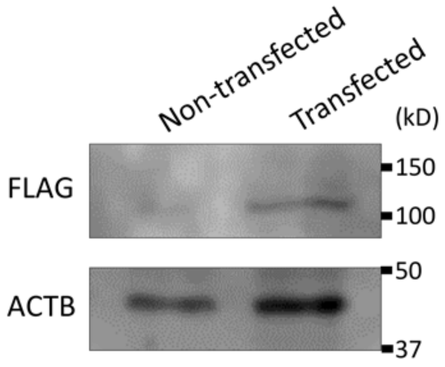


**Supplementary Figure 2. Confirmation of Cj-Cas9 expression by immunoblotting.**

A band with expectative molecular weight (about 118 kD) of Cas9 protein was observed in HEK293 cells. Beta-actin (ACTB) was shown as loading control.

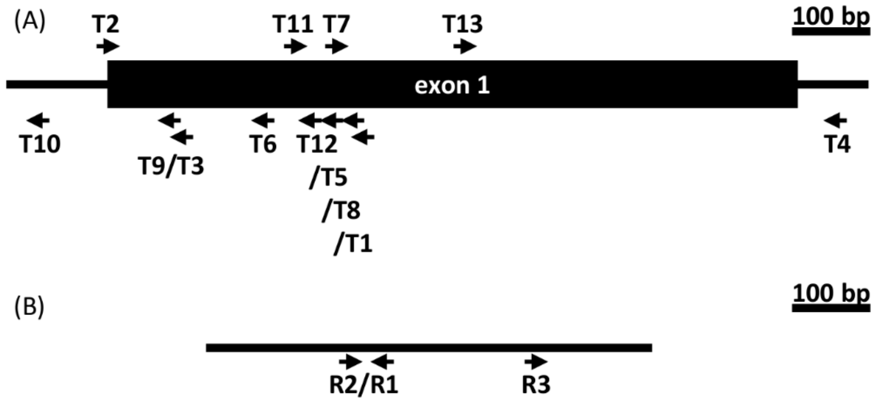


(C)

| gRNA | Target sequence | PAM | No. of blastocyst / 2-cell embryo (%) |
|---|---|---|---|
| T1 | GAGCACTGGCAGGTCCTATTA | TAAAACAC | 29/32 (90.6) |
| T2 | GGGGTTGCTGGAAAAGAAGTC | TGTGACAC | 24/26 (92.3) |
| T3 | GAGATCTGGAAACTCCACAGAA | GGCAATAC | 20/22 (90.9) |
| T4 | GAGCATGAAAATGTGGCTGC | TGAAGTAC | 24/26 (92.3) |
| T5 | GAGGGCCAGGACTCACGGTC | ATCCACCC | 19/20 (95) |
| T6 | GAAAGCTGGCCGCAGGGACTCC | CATCACCC | 18/18 (100) |
| T7 | GGGGTGGATGACCGTGAGT | CCTGGCCC | 30/30 (100) |
| T8 | CACTGGCAGGTCCTATTATA | AAACACAG | 16/16 (100) |
| T9 | GAAGGCAATACAAAACAGCC | AAGAACAT | 18/20 (90) |
| T10 | CATCAAATACTCTCAGCCTG | TTTTACAC | 23/25 (92) |
| T11 | CCTGCCAGGATATCCTTCTGTC | CAGTGCAC | 17/18 (94.4) |
| T12 | AAGGGGAACTGAGGTCCAGA | TGGTGCAC | 16/16 (100) |
| T13 | AAGTTTGGATTTGGGGGCCCA | AATTGTAC | 20/21 (95.2) |
| R1 | GAGAACTCCCAGAAAGGTAT | TGCAACAC | 24/26 (92.3) |
| R2 | AGTGGAGTAGGCGGGGAGAA | GGCCGCAC | 21/23 (91.3) |
| R3 | GTGGGAAGTCTTGTCCCTCCAA | TTTTACAC | 18/18 (100) |

**Supplementary Figure 3. Schematics of target loci of each gRNAs.**

(A) The gRNAs designed in Tyrosinase locus (Chr7: 87492422 to 87493521 in GRCm38.p4). (B) The gRNAs designed in Rosa26 locus (Chr6: 113075796 to 113076364 in GRCm38.p4). Each arrow indicates the target of gRNAs shown in Table 1 and Supplementary Figure 1. The direction of arrow means the 5’ to 3’ of gRNA recognition sequence. (C) Target sequences of each gRNAs and the developmental competencies of the microinjected embryos.

**Supplementary Table 1. Primer sets for synthesis of Cj-gRNA template vectors and ssODN for Chk2-Flag knock-in.**

| Target | Sequences |
|---|---|
| Fw primer for platform gRNA | 5’-GAAATTAACCCTCACTAAAGGAGAGACGATCGTCTCTGTTTTAGTCCCTGAAAAGGGACTAAAATAAAGA-3’ |
| Rv primer for platform gRNA | 5’-TTTAAAAGCGGTTTTAGGGGATTGTAACCCCGCAGAGTCCCGCAAACTCTTTATTTTAGTCCCTTTTCAG-3’ |
| ssODN for Chk2-Flag | 5’-GAACTGGAGGTGGAGGGTATGCCGAGCACAAAACGCCTGTCTGTGTGTGGGGCTGTGTTGGGATCCGATTACAAGGA TGACGACGATAAGATCTGAGCTTTGAATGTGAAAGAAATGTGCTTCCTTCCACGCTCCAATTGTCTTCTCCATCTTG-3’ |

**Supplementary Table 2. Primer sets for genomic PCR.**

| Target locus | Sequences | | |
|---|---|---|---|
| Tyrosinase target | Forward 1 | 5'- | TTATGCATTGAAGCAGTTCACC |
| | Reverse 1 | 5'- | TTCCAGATACCATAGGAGGTGC |
| | Forward 2 | 5'- | TTTGACAGTGGTGGAAACTGTCC |
| | Reverse 2 | 5'- | AGCAATGTTACTTCGCAGCAGAGC |
| Tyrosinase off-target-1 | Forward | 5'- | GGAGACAAAGTGCAGAGAAGAG |
| | Reverse | 5'- | GGGGGTCTGGTTGATAATGTTG |
| Tyrosinase off-target-2 | Forward | 5'- | GACATCAGCAACATTTTCCAAG |
| | Reverse | 5'- | CTTTGCAAGGTTCAACAGAGTG |
| Tyrosinase off-target-3 | Forward | 5'- | TATTCCCCTACACTGGGTCATC |
| | Reverse | 5'- | TGGCTAATGAGGGAGATAAAACCAG |
| Rosa26 locus | Forward | 5'- | ACGTTTCCGACTTGAGTTGC |
| | Reverse | 5'- | ATACTCCGAGGCGGATCAC |
| Chk2 | Forward | 5'- | AGGTGTGTGAGGAGGATTCG |
| | Reverse | 5'- | GTGCTGGGATTAAAGGAGTGG |

**Supplementary Table 3. On-target and off-target loci of *Tyrosinase*.**

| Target | Locus | Target sequences | Mutated/Total pups |
|---|---|---|---|
| Tyrosinase target | chr7: 87493021-87493158 | GAGCACTGGCAGGTCCTATTATAAAACAC | 20/20 |
| Off-target-1 | chr3: 61987856-61987993 | AGGCTCTGGCAGGGCCTCTTAGAAGACAC | 0/20 |
| Off-target-2 | chr8: 97686169-97686306 | AGGTTCTGGCAGGGCCTCTTAGGGGACAC | 0/20 |
| Off-target-3 | chr6: 8891204-8891341 | GACACCTGGCAGGTCCATTTAGAACACAC | 0/20 |
